# Effect of bentonite-activated carbon fillers addition to type II dental gypsum on its compressive strength, wettability, setting time, and setting expansion

**DOI:** 10.1186/s12903-026-08322-6

**Published:** 2026-04-22

**Authors:** Tamer M. Hamdy

**Affiliations:** Restorative and Dental Materials Department, Oral and Dental Research Institute, National Research Centre (NRC), Dokki, Giza, 12622 Egypt

**Keywords:** Dental plaster, Improved stone, Gypsum products, Bentonite-activated carbon, Compressive strength

## Abstract

**Background:**

Dental plaster, dental stone, and improved stone are commonly used gypsum products for dental molds. While improved stone is preferred for final molds due to its strength, dental plaster is cheaper but has low compressive strength, limiting its use to temporary models.

**Objective:**

To evaluate the compressive strength, wettability, setting time, and setting expansion of Type II dental plaster modified with 10 wt% calcium bentonite and activated carbon fibers.

**Methods:**

The modified dental plaster had significantly higher compressive strength (43.5 ± 1.4 MPa) than the conventional dental plaster (24.3 ± 1.3 MPa) and improved stone (36.1 ± 0.4 MPa) (P 0.05). There were no statistical differences between groups regarding initial or final setting times (*P* > 0.05).

**Results:**

The modified dental plaster displayed significantly higher compressive strength (43.5 ± 1.4 MPa) compared to conventional dental plaster (24.3 ± 1.3 MPa) and improved stone (36.1 ± 0.4 MPa) (*P* < 0.001). Wettability was obviously improved, with a contact angle of 0° ± 0, compared to 99.3° ± 0.2 for conventional plaster and 93.6° ± 0.6 for improved stone (*P* < 0.001). Setting expansion was significantly decreased in the modified group (0.05% ± 0.01) compared to conventional plaster (1.36% ± 0.01), with no significant difference from improved stone (0.07% ± 0.01) (*P* > 0.05). No significant differences were shown in initial or final setting times among the groups (*P* > 0.05).

**Conclusion:**

Incorporation of 10 wt% Bentonite–activated carbon fillers markedly improve the compressive strength and wettability of Type II dental plaster, reducing setting expansion without compromising setting time. Modified material has the potential to be a tooth and plaster accelerated cement automatic operation, which is a more economical solution than traditional Type IV improved stone for dental model purposes.

## Background

Models of oral tissues play a crucial role in dentistry for evaluating, treating, and creating indirect restorations [1, 2]. Gypsum products are widely used in dentistry to make dental models and dies. These models and dies are then used for the construction of indirect dental restorations like crowns and bridges. The American Dental Association (ADA) categorizes dental gypsum products into five distinct types based on their properties and applications: impression plaster (type I), dental plaster (type II), dental stone (type III), improved stone (type IV), and high expansion improved stone (type V) [3].

Type II dental gypsum (model plaster) is mainly utilized for making initial study models and diagnostic casts. Type III dental gypsum (dental stone) is more robust and long-lasting than model plaster, making it ideal for producing working models, orthodontic models, and castings that demand higher strength and precision. Type IV dental gypsum (improved stone) is known for its exceptional strength, accuracy, and durability, making it a preferred option over traditional dental stone for various dental procedures. It is frequently utilized in the creation of fixed prosthodontic restorations, crowns, bridges, and implant models as it produces highly precise models of the patient’s teeth and oral structures. These models are essential for designing and constructing dental restorations that ensure an optimal fit and function [4, 5].

All these gypsum products are made of the same chemical, which is calcium sulfate hemihydrate (CaSO_4_.2H_2_O). The only differences are in the size and shape of the particles, which depend on their production. As a result, each type exhibits different mechanical properties [6–8].The process of manufacturing dental plaster relies on the quick and direct heating of calcium sulfate dihydrate in open air, resulting in a powder made up of weak, irregularly shaped porous particles. In contrast, improved stone is produced by calcining in a calcium chloride solution, which yields more uniform, denser, smaller, and stronger particles [9].

Type II dental gypsum, also known as dental plaster, consists of β-hemihydrate particles. The primary disadvantages of using dental plaster include its lower mechanical properties, along with higher setting expansion during the setting process. As a result, dental plaster is primarily utilized for temporary (study) models rather than final ones. In contrast, type IV dental gypsum is made up of α-hemihydrate particles and is frequently used to produce final dental models due to its superior mechanical properties, and reduced setting expansion [10].

The optimal properties for gypsum products used for final model materials are high level of mechanical properties to withstand the manipulation forces during laboratory procedures. In addition, poor setting time, and predictable setting expansion are required to obtain a precise fit for the final dental restoration [11, 12].

Bentonite is a type of clay that is rich in silica (SiO_2_). They are classified according to their dominant elements other than silica, including potassium (K), sodium (Na), calcium (Ca), Magnesium (Mg), and aluminum (Al). The initial application of bentonite clay in dentistry was due to its role as a detoxifying agent, which enhances oral health [13]. Various studies have shown that adding clay as a filler to dental materials enhances their mechanical properties [14, 15]. Adding bentonite to materials can provide significant advantages in terms of strength and durability [16, 17]. The study conducted by Iacob et al. about the impact of adding bentonite as a filler in composites showed that it improves mechanical strength [18]. Furthermore, it is shown that the incorporation of activated carbon fibers into composite materials enhance their mechanical strength, improve their thermal stability, and lower the linear coefficient of expansion [19–21]. The combination of bentonite clay and activated carbon fiber could improve the effectiveness of the filler, as clays have been utilized as binders in carbon mixtures to provide strength to the resulting carbon structure [22].

Adding fillers to gypsum products can greatly change their properties and applications [9, 23]. Little studies have been made to improve the mechanical properties of gypsum products by adding various filler additives [9, 24]. Earlier research enhanced the strength of dental plaster and decreased its setting expansion by adding 10% by weight of aluminum oxide nanoparticles [24]. Additionally, improving dental plaster is done by adding Arabic gum and calcium hydroxide filler [25]. Furthermore, titanium oxide may be incorporated to enhance mechanical properties and minimize setting expansion [26].

The aim of this *vitro* study is to modify type II dental gypsum with manual blending of 10 wt% calcium bentonite clay/activated-carbon fiber (bentonite-activated carbon) fillers to improve their compressive strength, wettability, and decrease their setting expansion. The null hypothesis was that the modification of type II dental gypsum by addition of 10 wt% bentonite-activated carbon fillers would not affect the compressive strength, wettability, setting time, and expansion compared to the conventional non-modified type II and type IV dental gypsum groups.

## Methods

### Calculation of the sample

Sample size estimation was based on data obtained from a previous studies [24, 27]. Sample size was calculated by G*Power software version 3.1.9.7 (Heinrich Heine University Duesseldorf, Duesseldorf, Germany) with an alpha level set at 0.05 and a power of 85%, the minimum required sample size was determined to be *n* = 10 specimens per subgroup for each tested property.

### Study design

**Fig. 1:**
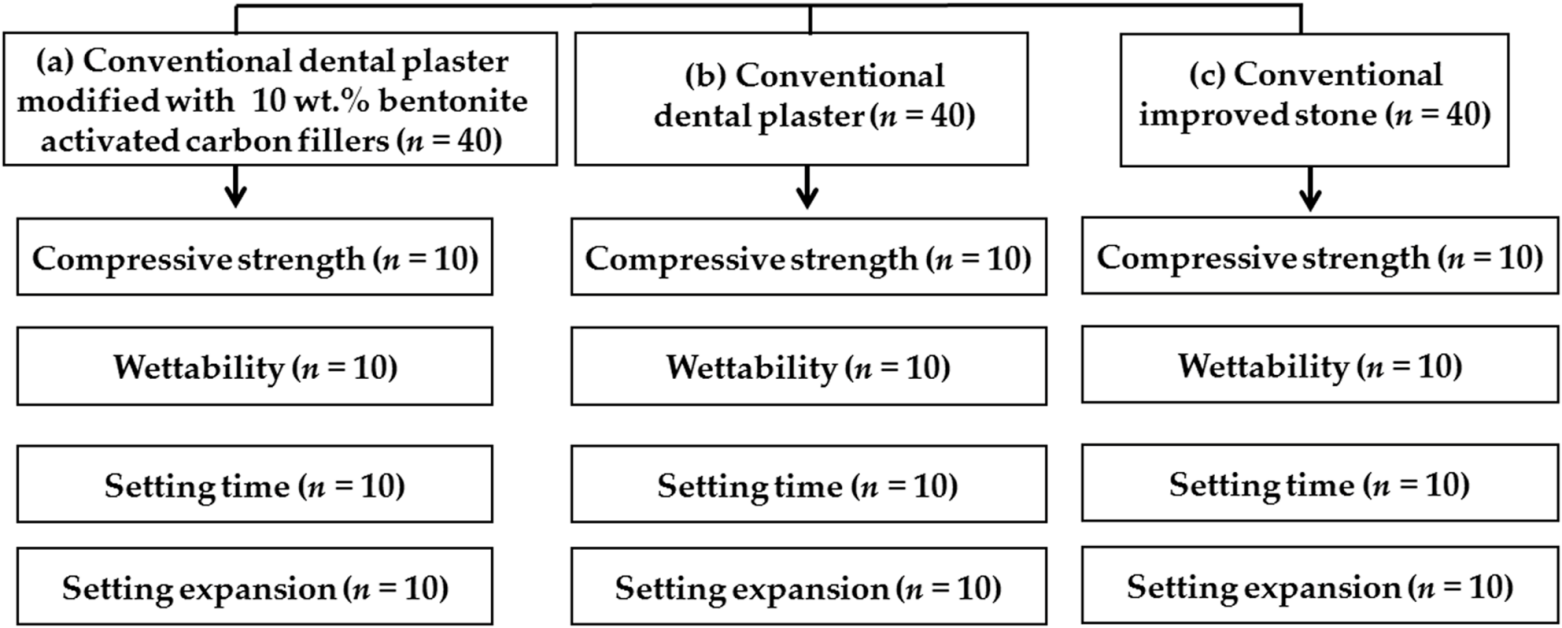
Flowchart illustrating the different groups of specimens

Three main groups were included in this experimental laboratory study: (a) conventional dental plaster modified with 10 wt% bentonite-activated carbon fillers, (b) conventional dental plaster, and (c) conventional improved stone. A total of 120 specimens were prepared. They were standardized and evenly distributed in three main groups of 40 specimens according to each type of used material. These were divided into four subgroups of specimens (*n* = 10) for examination of compressive strength, wettability, setting time, and setting expansion test. Figure 1 represents the distributions of groups according to each material used and the performed test.

### Manual incorporation and blending of fillers

Bentonite–activated carbon fillers were mixed with dental plaster powder using a standardized dry mixing protocol. The fillers (10 wt% of total powder weight) were accurately weighed using a digital analytical balance (Adam Equipment 4-digit precision weighing balance, Adam Equipment Inc., Oxford, UK) and gradually added to the plaster powder. In a clean and dry glass container, the mixture was homogenously mixed using circular folding motion with stainless-steel spatula for 5 min. Subsequently, the blended powder was sieved through a 100 μm mesh in order to remove agglomerates and promote better dispersion of the particles and further mixed for another 2 min. Homogeneity of the powder mixture was verified by visual inspection, ensuring uniform color and absence of visible particle clusters. All procedures were conducted under controlled laboratory conditions at room temperature in a dry environment to prevent premature hydration.

### Preparation of the specimens

Conventional dental plaster groups (negative control) were prepared by mixing dental plaster (Dental Plaster Type II, Garreco, USA) with water. The modified dental plaster groups were prepared by manual incorporation of 10 wt% bentonite-activated carbon fillers purchased from (USA Lab Equipment, Livonia, Michigan, USA) to the conventional dental plaster powder before water mixing. The improved stone groups (positive control) were prepared by mixing improved stone powder (Royal Rock Type IV, Garreco, USA) with water. For all groups, the powder was mixed with distilled water according to standardized water-to-powder ratios: type II dental plaster: 0.50 mL/g, modified plaster: 0.50 mL/g, type IV improved stone: 0.30 mL/g. Mixing was performed using a plaster vibrator (Technoflux, Barcelona, Spain) for 30 s to obtain a homogeneous, bubble-free slurry. The mixture was then poured into prefabricated molds corresponding to each test.

### Chemical composition analysis of the fillers

The elemental composition of the bentonite–activated carbon filler mixture (prior to incorporation into gypsum) was determined using X-ray fluorescence (XRF) analysis (X-MET3000TXR, Oxford Instruments GmbH, Germany) to detect and confirm their chemical composition [28, 29]. The analysis was operated at an accelerating voltage of approximately 40–50 kV with automatic current adjustment. Calibration was performed using factory pre-set calibration standards prior to measurement. The analysis provided semi-quantitative elemental composition expressed as weight percentages of the detected oxides.

### Compressive strength analysis

Compressive strength was evaluated according to ANSI/ADA Specification No. 25. Cylindrical specimens (3 mm diameter × 6 mm height) were prepared (*n* = 10 per group). A Teflon mold, measuring 3 mm in diameter and 6 mm in height, was created and filled with freshly mixed gypsum products. The specimens were removed from the mold after 1 h and kept for 24 h at 37 °C with 95 ± 5% relative humidity in an incubator (CBM, S.r.l. Medical Equipment, 2431/V, Cremona, Italy). The compressive strength test was carried out using a Universal Testing Machine (Shimadzu Autograph AG-X plus, Kyoto, Japan) at a crosshead speed of 1 mm/min, utilizing a 5KN load cell [3]. Compressive strength (MPa) was calculated using the formula: Compressive strength = F/A; Where: F = maximum load at fracture (N), A = cross-sectional area (mm²).

### Wettability analysis

The contact angle was measured to assess the wettability of each material tested. Disc-shaped specimens (20 mm diameter × 2 mm thickness) were prepared (*n* = 10) using a stainless-steel circular mold, which was then covered with a glass plate and allowed to harden at room temperature under a load of 1 kg. The specimen surfaces were cleaned and dried prior to testing. A 5 µL distilled water droplet was applied using a droplet analysis system (SmartDrop, Femtofab, Korea), and the contact angle was measured immediately after droplet placement using the sessile drop method to assess the hydrophilicity of the samples [30, 31]. Three measurements were recorded at different locations on each specimen, and the mean value was calculated.

### Setting time analysis

Initial and final setting times were determined using a Gillmore needle apparatus (Humboldt MFG., Norridge, IL, USA) according to ADA standards. Measurements were conducted under controlled environmental conditions (23 ± 2°C) [32]. The initial setting time test was determined using A needle weighing 113.4 g and having a tip diameter of 2.12 mm. The final setting time was measured using A needle weighing 453.6 g and having a tip diameter of 1.06 mm. Measurements were repeated at regular intervals until no visible indentation was observed.

The Gilmore needle was located vertically against the sample with its tip in gently touching the surface. Once in place, the needle was released, allowing it to penetrate the sample. The time of the needle for penetrating the sample was 15 s. After that, the needle was pulled out, and any gypsum that attached to the tip was wiped off with tissue paper, getting it ready for the next penetration area. The needle was used to make several penetrations around the sample to explore different areas. Each time, the needle was removed after 15 s, which meant that each penetration session lasted a total of 30 s. This continued until the needle could no longer leave a mark on the gypsum surface. Once the needle could no longer make penetration, the test moved on to test the final setting time, using the same method as for the initial setting time [5].

### Setting expansion analysis

Linear setting expansion was measured using an extensometer with a micrometric dial gauge (Novanna limited, Great Barton, UK). Measurements were recorded 2 h after mixing, based on established protocols in previous studies. Expansion values were expressed as percentage change relative to the original length [33].

### Statistical analysis

Data were analyzed using SPSS software (version 27.0, IBM Corp., Chicago, IL, USA). Normality of data distribution was assessed using the Shapiro–Wilk test. Comparisons among groups were performed using one-way ANOVA followed by Tukey post hoc test was used (α = 0.05). Statistical significance was set at *P* < 0.05. Effect size (η²) was calculated to assess the magnitude of differences.

## Results

### Chemical composition analysis of the fillers

The chemical composition of the bentonite-activated carbon fillers was determined using XRF technique as represented in Table 1. The XRF results showed bentonite composition with high percentage of silica (56.7%) and calcium oxide (16.83%), with a small amount of activated carbon fibers (7%).

**Table 1:** Chemical composition of the filler mixture determined by XRF analysis

Ingredient	Mass Percentage (%)
Silicon dioxide (silica)	56.7
Calcium Oxide	16.83
Ferric Oxide	7.65
Magnesium Oxide	7.57
Aluminum Oxide	2.33
Sodium Oxide	0.29
Potassium Oxide	1.34
Phosphorus pentoxide	0.29
Carbon fibers	7

### Compressive strength analysis results

Table 2 shows the results of the mean compressive strength values. The modified dental plaster showed the highest compressive strength, followed by conventional improved stone followed by conventional dental plaster (43.5, 36.1 and 24.3 MPa respectively, (*P* = 0.001*). Effect size analysis revealed a very large effect (η² = 0.97), indicating a strong influence of material modification.

**Table 2:** Compressive strength mean and standard deviation for all groups

Gypsum	Compressive strength ± SD (MPa)	*P* value
Modified dental plaster	43.5 ^a^±1.4	0.001*
Conventional dental plaster	24.3 ^c^±1.3	
Conventional improved stone	36.1 ^b^±0.4	

^*^Difference small letters in the same column denotes a significant difference (*P* < 0.05)

### Wettability analysis results

**Fig. 2:**
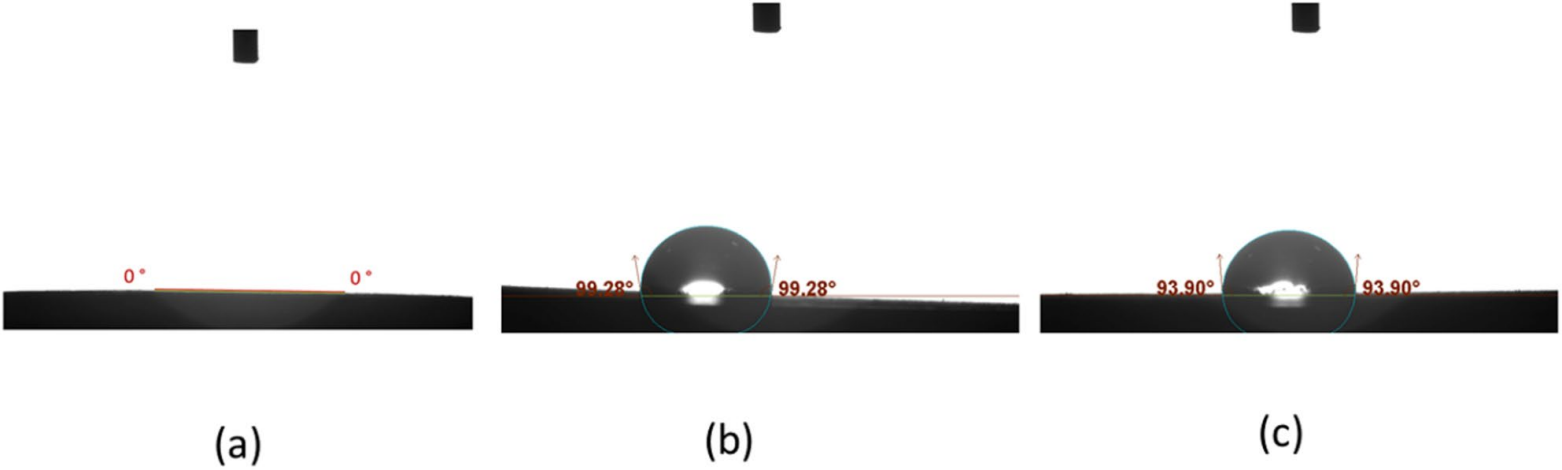
Representative images of water droplets on different groups of the specimens: **a** modified dental plaster, which showed a zero contact angle corresponding to maximum hydrophilicity; **b** conventional dental plaster, which showed the highest contact angle corresponding to the lowest wettability and hydrophilicity; **c** conventional improved stone, which showed a high contact angle corresponding to low wettability and hydrophilicity

The mean, standard deviation values for contact angle (°) was demonstrated in Table 3 and Fig. 2. The modified dental plaster showed the least contact angle i.e. highest wettability followed by conventional improved stone, followed by conventional dental plaster (0, 93.6 and 99.3 °) respectively. The effect size was extremely large (η² = 0.99), confirming a substantial impact on surface properties.

**Table 3:** Contact angle mean and standard deviation for all groups

Gypsum	Contact angle ± SD (°)	*P* value
Modified dental plaster	0^c^±0	0.001*
Conventional dental plaster	99.3^a^±0.2	
Conventional improved stone	93.6^b^±0.6	

^*^Difference small letters in the same column denotes a significant difference (*P* < 0.05)

### Setting time analysis results

The mean, standard deviation values for the initial and final setting time (min.) was demonstrated in Table 4. There was no significant difference between the setting time of the three tested groups, either the initial or the final setting time, with a negligible effect size (η² = 0.02).

**Table 4:** Initial and final setting time mean and standard deviation for all groups

Gypsum	Initial setting time ± SD (min.)	Final setting time ± SD (min.)
Modified dental plaster	8.17 ± 0.1	14.9 ± 0.3
Conventional dental plaster	8.27 ± 0.1	15.1 ± 0.1
Conventional improved stone	8.2 ± 0.1	14.6 ± 0.3
*P* value	0.9	0.5

### Setting expansion analysis results

The mean, standard deviation values for the setting expansion (%) was presented in Table 5. The conventional dental plaster showed the highest significant setting expansion (1.36%), followed by conventional improved stone (0.07%), followed by modified dental plaster (0.05%). There was no statistically significant difference between modified dental plaster and improved stone (*P* > 0.05). The effect size was very large (η² = 0.98).

**Table 5:** Setting expansion mean and standard deviation for all groups

Gypsum	Setting expansion ± SD (%)	*P* value
Modified dental plaster	0.05 ^b^±0.01	0.001*
Conventional dental plaster	1.36 ^a^±0.01	
Conventional improved stone	0.07^b^±0.01	

^*^Difference small letters in the same column denotes a significant difference (*P* < 0.05)

## Discussion

The advancement of materials and techniques that improve both impressions and cast models is crucial for achieving clinical success. Creating an accurate casting relies on several factors, such as the type of dental impression materials and technique used, the precision of the model and die materials, and the processes involved in waxing and casting [12].

The compressive strength test reveal details regarding the load-bearing ability and structural integrity of the gypsum [34]. Moreover, the initial and final setting time findings offered important insights into the workability of the gypsum mixtures and their setting properties. Reducing the setting time of dental gypsum is essential for the prompt removal of impressions. This helps to prevent dimensional changes and ensures accurate modeling [5]. Additionally, achieving low setting expansion along with adequate compressive strength are the two key requirements for a die material [5, 35, 36].

According to the ADA specification no. 25 [3], type IV gypsum (conventional improved stone) minimum compressive strength is about 35 MPa, the initial setting time is about 5 ± 1 min. and the final setting time is about 12 ± 4 min. Moreover, it should provide a dimensional stability of up to 0.1%. The addition of a filler greatly influences the compressive strength, setting time, and dimensional stability [35].

Wetting of a surface is the extent to which a drop spreads across a solid surface. The smaller the contact angle, the greater the wettability, and the greater the hydrophilicity. The surface reproduction and the subsequent accuracy of the indirect dental restorations is improved by increasing of the hydrophilicity of the materials, which is determined by decreasing the contact angle [37].

In the current study 10 wt% bentonite-activated carbon fillers have been used to indicate whether there is an improvement in compressive strength, wettability, setting time, and setting expansion of type II dental gypsum or not. The null hypothesis was rejected as the modified type II dental gypsum exposed an increased compressive strength and wettability, while it provided a reduced setting expansion compared to conventional non-modified type II and type IV dental gypsum. However, there is no effect on setting time for all tested groups.

The XRF analysis is a non-destructive method used for determining the elemental analysis of the materials [38]. The chemical composition of the bentonite-activated carbon fillers was verified. The XRF spectra reveal the high weight% of silica and calcium oxide with a small amount of carbon fiber [28, 29].

Increasing the compressive strength dental gypsum is highly significant for dental applications, especially in the fabrication of precise and durable working models and dies for indirect restorations [34]. Higher compressive strength reduces the risk of die or model fracture during laboratory procedures, maintain the dimensional stability, ensuring accurate reproduction of oral structures and precise fitting of dental restorations, improves the resistance to abrasion, and supporting the longevity of the models [34, 39].

The study results on compressive strength revealed that the modified group, which included bentonite-activated carbon fillers in the conventional type II dental gypsum, demonstrated a significant enhancement in the compressive strength compared to the unmodified groups. The high compressive strength observed in the modified group may be attributed to the filler reinforcement effect, reduction in internal porosity, and improved particle packing, which collectively enhance load distribution within the material [40]. Moreover, the fillers may be filling up the voids and decreasing the number and size of voids [41]. In addition, the enhanced mechanical properties of bentonite activated carbon fillers may be attributed to the reinforcement effect of the Carbone fiber. In addition, The calcium bentonite clay has the potential to be partially exfoliated into nanometric platelets with a high aspect ratio, which can subsequently be dispersed within the matrix [42].

These results agree with a study conducted by Atai et al. Who reported that combination of nanoclay to dental adhesives can improves their mechanical properties [40]. Furthermore, the study conducted by Waqas et al. provided that the incorporation of bentonite as a filler by (10 wt%) exhibited considerable improvement in the strength and durability characteristics of geopolymer concrete [43]. The results of this study align with those reported by Hamdy et al., who found that dental plasters enhanced with alumina nanoparticles exhibited a notable increase in compressive strength [24]. Moreover, it come in agreement with the study performed by Fode et al. who found that the addition of 20% bentonite fillers can improve the mechanical properties of the cement composite materials including Portland cement [44].

Increasing the wettability in dental gypsum is essential for producing accurate, high-quality dental casts by improving the detail reproduction, minimizing air entrapment, and enhancing the adaptation of models and dies to the surface of the dental impressions [45]. The water contact angle provides an indication about the surface permeability of the materials and reveals their hydrophilic properties [46]. The zero-contact angle observed in the modified group indicates complete wetting behavior, which may be attributed to the highly hydrophilic nature of bentonite. This behavior suggests enhanced surface energy and improved liquid spreading ability [47]. This behavior can be explained by the hydrophilic nature of bentonite, which increases surface energy and promotes liquid spreading. Clinically, improved wettability enhances the interaction between gypsum products and impression materials, leading to better detail reproduction [48].

All tested materials exhibited setting times within acceptable ADA limits, indicating their suitability for clinical application. No significant differences in setting time were observed among the groups, indicating that the modification does not adversely affect handling properties. This is an important factor for clinical applicability, which could be due to the small amount of the of fillers used. Using small amounts of bentonite fillers can enhance workability without significantly impacting the setting time, which means the setting process stays within acceptable limits for practical applications. However, this is influenced by the concentration and type of bentonite used [44].

Reducing the setting expansion of dental gypsum is vital for producing accurate, stable, and well-fitting dental restorations. The most effective approaches involve adding specific additives while maintaining optimal strength and stability [36]. The smaller dimensional changes observed in the modified group can be attributed to the lower setting expansion values, which result from adding bentonite-activated carbon fillers on expense of calcium sulfate dihydrate powder. This leads to a decrease in the amount of calcium sulfate dihydrate crystals produced, which in turn reduces the outward thrust action of the formed crystals, ultimately resulting in less linear expansion [9]. Moreover, the reduction in the expansion of the modified groups may be due to the effect of bentonite-activated carbon fillers, which lead to the diminishing of voids volume [49].

In summary, the present study reported that the modification of type II dental gypsum by addition of 10 wt% bentonite-activated carbon fillers exhibited an increase in the compressive strength, wettability, decrease in the initial and final setting times, with a reduction in setting expansion that exceed the minimum requirement stated by the ADA specification no. 25 for type IV gypsum [3]. Overall, the findings suggest that the modified dental plaster can serve as a cost-effective alternative to Type IV improved stone, offering enhanced performance without compromising usability.

This study did have some limitations, as the findings did not explore the effect of different particle sizes and concentrations of the fillers. It is recommended that future studies investigate the effects of including bentonite-activated carbon fillers at higher concentrations and with different powder-to-water ratios. Additionally, confidence intervals were not calculated and are considered a limitation of the study.

## Conclusions

Incorporating 10 wt% bentonite activated carbon fillers into conventional dental plaster may enhance its compressive strength and wettability while lowering its setting time and setting expansion. Therefore, this modified dental plaster with bentonite activated carbon at the chosen concentration could serve as a viable alternative to conventional improved stone.

## Data Availability

The datasets generated during and/or analyzed during the current study are not publicly available due to institutional policy but are available from the corresponding author on reasonable request.

## Abbreviations

Bentonite-activated carbon: calcium bentonite clay / activated carbon fiber
ADA: American Dental Association
XRF: X-ray fluorescence
ANSI: American National Standards Institute
SPSS: Statistical Package for the Social Sciences
